# Supplementary material for: Constructing 3D Skeleton on Commercial Copper Foil via Electrophoretic Deposition of Lithiophilic Building Blocks for Stable Lithium Metal Anodes
Source: Nanomaterials (Basel). 2023 Apr 18;13(8):1400. doi: 10.3390/nano13081400 (PMC10146236; doi:10.3390/nano13081400)
Supplement: Supplementary file 1 [file nanomaterials-13-01400-s001.zip › nanomaterials-2322010-supplementary.pdf]

Supplementary Materials

Constructing 3D Skeleton on Commercial Copper Foil via Electrophoretic Deposition of Lithiophilic Building Blocks for Stable Lithium Metal Anodes

Yun Jiang [1], Wenqi Zhang [2], Yuyang Qi [2], Yuan Wang [2], Tianle Hu [1], Pengzhang Li [1], Chuanjin Tian [1], Weiwei Sun [3], and Yumin Liu [1,*]

[1] Institute of New Energy Materials and Devices, School of Materials Science and Engineering, Jingdezhen Ceramic University, Jingdezhen 333403, China
[2] Institute for Interdisciplinary Research (IIR), Jianghan University, Wuhan 430056, China
[3] College of Aerospace Science and Engineering, National University of Defense Technology, Changsha 410073, China
[*] Correspondence: ymliu@jhun.edu.cn

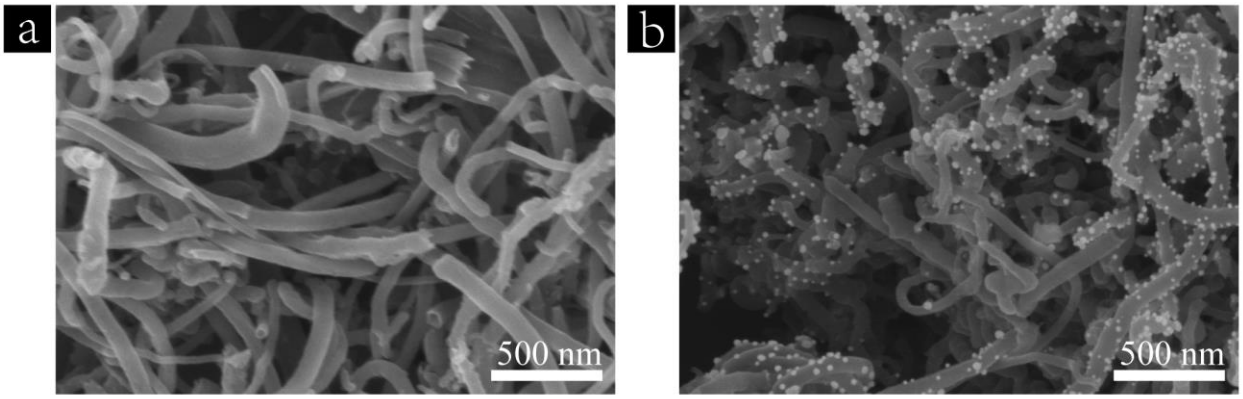

Figure S1. The SEM images of (a) CNTs and (b) as-prepared Au@CNTs.

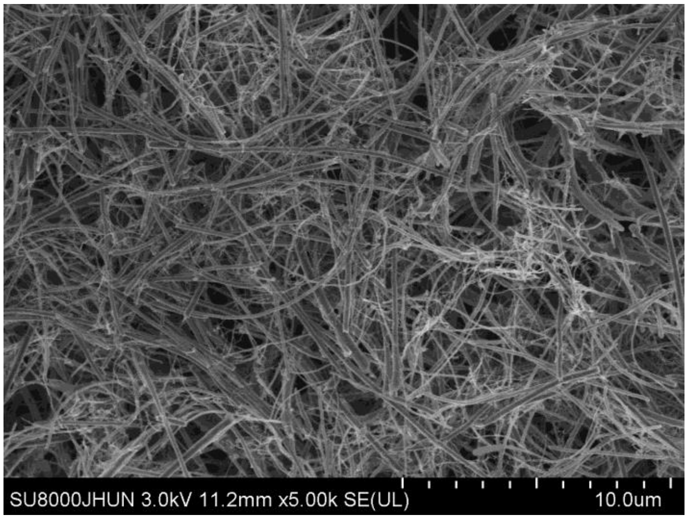

Figure S2. Top-view SEM image of the obtained CNTs@Cu foil.

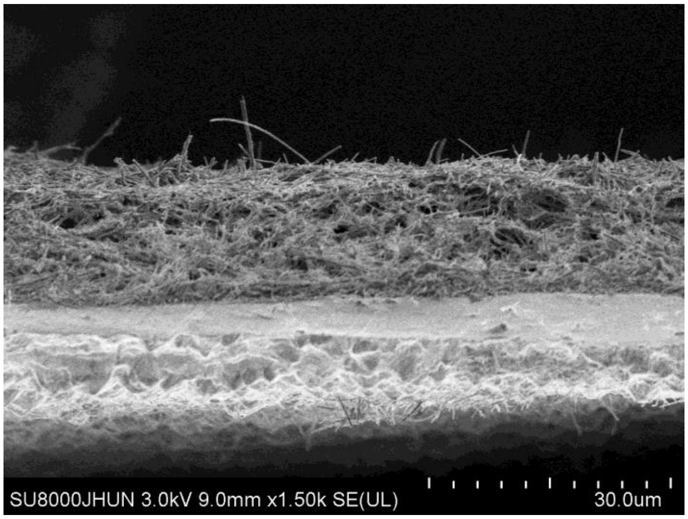

Figure S3. Cross-sectional SEM image of the obtained CNTs@Cu foil.

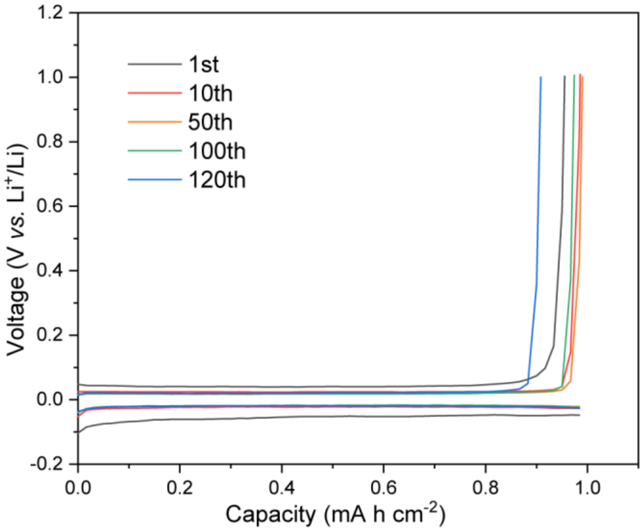

Figure S4. Voltage profiles of Cu foil under different cycles at a current density of 1 mA cm^{-2} with an areal capacity of 1 mA h cm^{-2}.

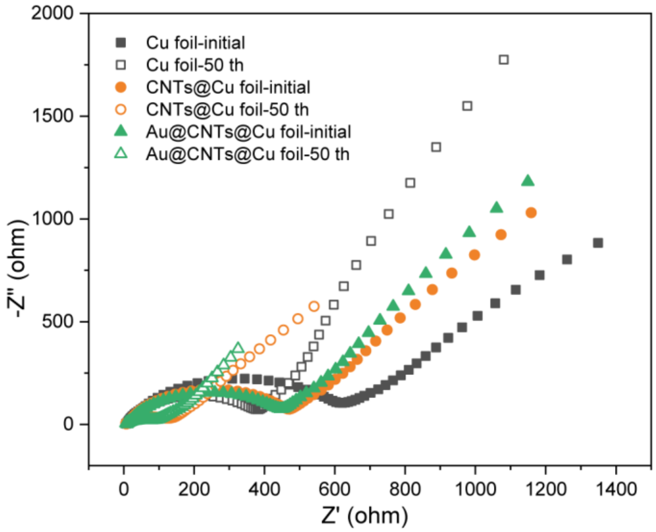

Figure S5. The Nyquist plot of the electrochemical impedance spectroscopy (EIS) spectra of Cu foil, CNTs@Cu foil, and Au@CNTs@Cu foil before initial cycle and after 50 cycles at 1 mA cm^{-2} with 2 mAh cm^{-2}.

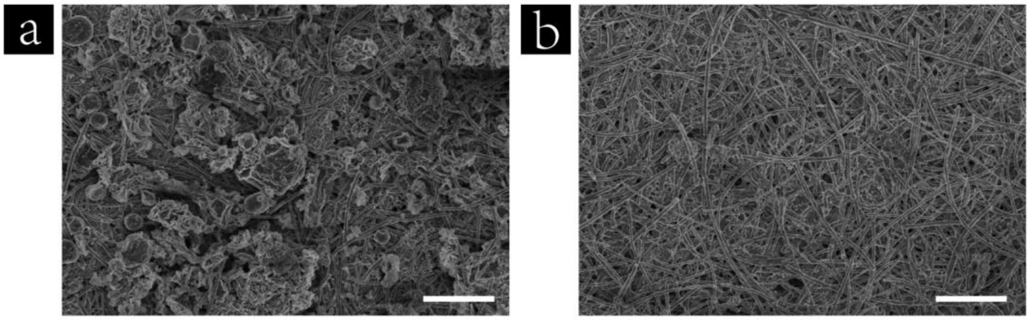

Figure S6. Top-view SEM images of stripped (a) CNTs@Cu foil and (b) Au@CNTs@Cu foil after 50 cycles.

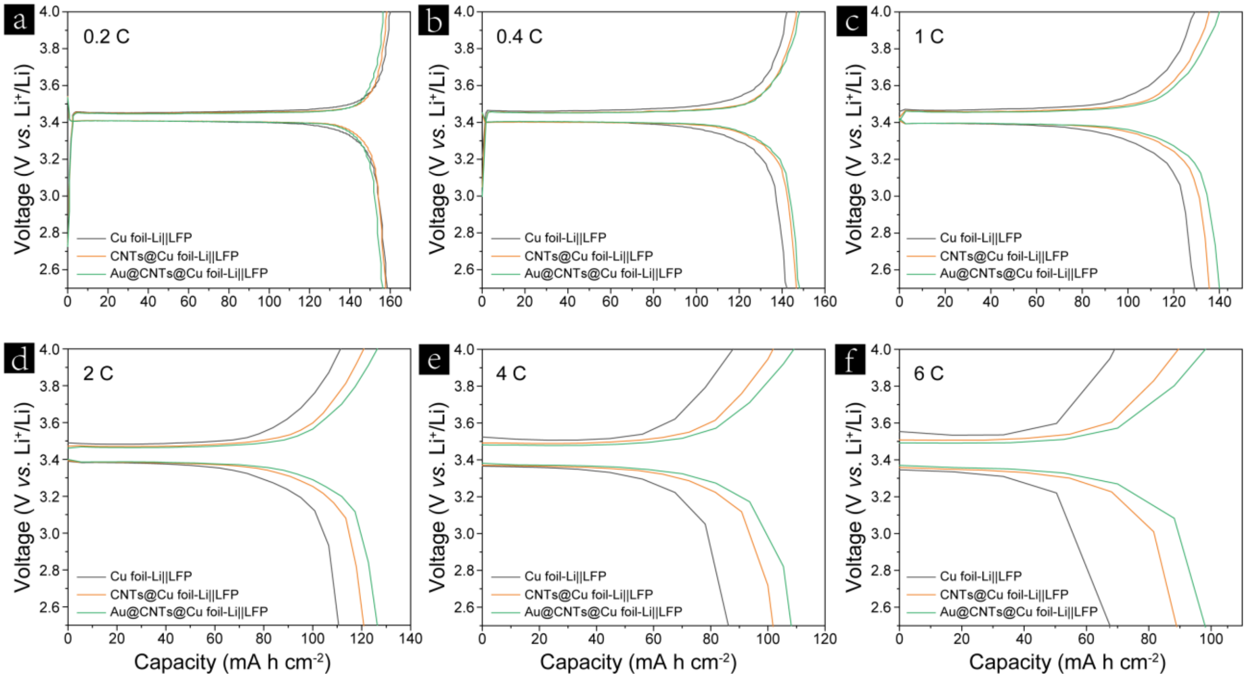

Figure S7. Voltage profiles of the full cells with the configuration of Cu foil-Li||LFP, CNTs@Cu foil-Li||LFP, and Au@CNTs@Cu foil-Li||LFP at different current rates.
